# Heparin Mimetics and Their Impact on Extracellular Matrix Protein Assemblies

**DOI:** 10.3390/ph16030471

**Published:** 2023-03-22

**Authors:** Fabian Heide, Manuel Koch, Jörg Stetefeld

**Affiliations:** 1 Department of Chemistry, University of Manitoba, Winnipeg, MB R3T 2N2, Canada; 2 Institute for Experimental Dental Research and Oral Musculoskeletal Biology, Center for Biochemistry, Medical Faculty, University of Cologne, 50931 Cologne, Germany; manuel.koch@uni-koeln.de

**Keywords:** heparan-sulfate-binding proteins, extracellular matrix organization, protein multimers, protein aggregation, heparin mimetics, protein–ligand interactions, clinical drug development

## Abstract

Heparan sulfate is a crucial extracellular matrix component that organizes structural features and functional protein processes. This occurs through the formation of protein–heparan sulfate assemblies around cell surfaces, which allow for the deliberate local and temporal control of cellular signaling. As such, heparin-mimicking drugs can directly affect these processes by competing with naturally occurring heparan sulfate and heparin chains that then disturb protein assemblies and decrease regulatory capacities. The high number of heparan-sulfate-binding proteins that are present in the extracellular matrix can cause obscure pathological effects that should be considered and examined in more detail, especially when developing novel mimetics for clinical use. The objective of this article is to investigate recent studies that present heparan-sulfate-mediated protein assemblies and the impact of heparin mimetics on the assembly and function of these protein complexes.

## 1. Introduction

Intercellular signaling and correct structural organization are largely mediated by the extracellular matrix, which functions to organize macromolecules in a local and temporal manner. This is necessary for multicellular life [1,2], and as such, alterations to the extracellular matrix structures and functions can cause an abundance of diseases [3]. Key components that assemble macromolecules such as proteins are the polysaccharides heparan sulfate and heparin. Heparan sulfates are a class of sulfated glycosaminoglycans (GAGs) that form large structural networks around cellular surfaces that can bind to heparan-sulfate-binding motifs on proteins [4]. These interactions are mostly mediated by the sulfation pattern of the ligand [5], which can vary depending on the cell type and state [6]. The unique combination of chemical and physical properties of heparan sulfates and heparins, such as the presence of negative charges and the flexibility of the sugar chains, allows them to bind to different molecules and therefore mediate various physiological processes. These include blood coagulation [7] and cellular signaling for inflammation, proliferation, differentiation and apoptosis [8,9,10,11]. Due to the large impact of these ligands on cellular homeostasis, it is important to understand the mechanisms underlying interaction with other molecules to allow for the development of targeted therapies for a wide range of diseases.

For this purpose, heparin-based drugs including heparin-mimicking drugs are actively being developed [12,13,14]. Heparin-mimicking drugs are chemicals that mimic the structure and possibly the function of naturally occurring heparan sulfate chains. These mimetics offer additional advantages over heparin drugs isolated from crude extracts, and research developments have recently been focused to expand our repertoire [15]. However, it is important to note that these drugs can disrupt the organization and function of larger protein complexes that depend on heparan sulfates for stability [16,17]. In addition, soluble heparin can cause artificial aggregation of proteins [18,19] that limit their function as drugs. These disruptions could lead to unexpected and potentially harmful side effects, especially in cases where the targeted protein complexes play a vital role in maintaining normal cellular physiology. Therefore, it is important to understand the role of heparin drugs in the context of protein multimer formation in order to limit negative side effects and effectively develop therapeutic alternatives for clinical settings.

## 2. The Role of Heparan Sulfates in Cellular Signaling and Organization

**Figure 1:**
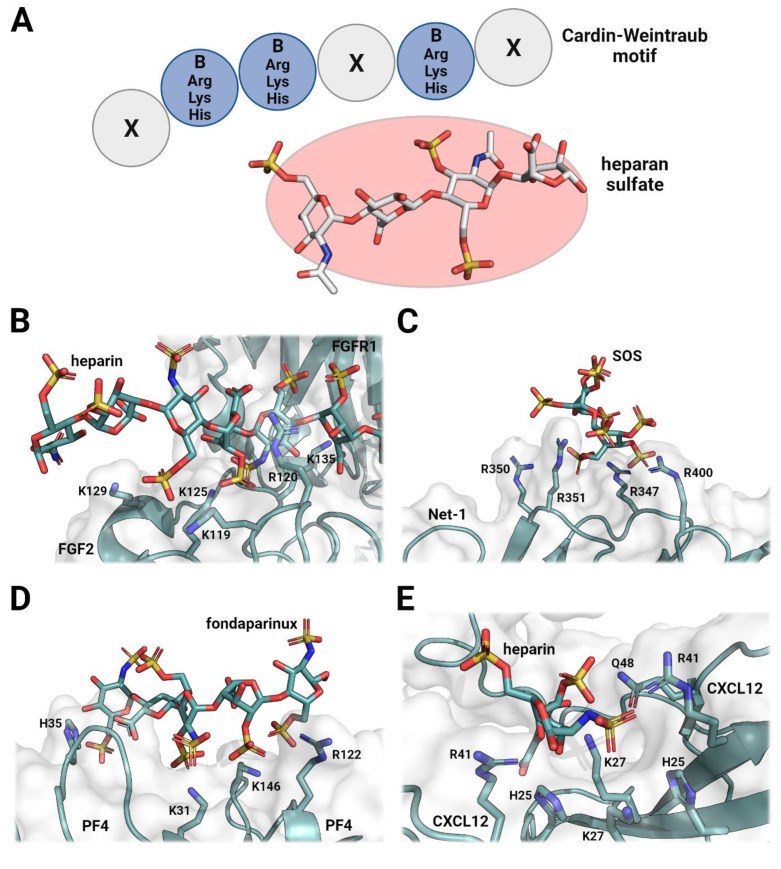
Structural examination of heparan-sulfate-based ligands and their respective protein binding sites. Protein–ligand interactions mostly occur over arginine and lysine residues, which form various protein surface interfaces. Some other contributing residues include histidine and glutamine, which stabilize the complexes through additional electrostatic or hydrogen-bonding interactions. This results in amino acid variations, which have drastic effects on the affinity and specificity of heparan sulfate or heparin ligands. (**A**) Schematic of the XBBXBX Cardin–Weintraub motif and its potential interactions with a heparin ligand. Protein–ligand interaction mostly occurs through electrostatic forces between positively charged (blue) amino acid side chains and negatively charged (red) sulfate groups on heparin. (**B**) Fibroblast growth factor 2 (FGF2) in complex with fibroblast growth factor receptor 1 (FGFR1), which is mediated by a long-chain heparin ligand (PDB ID: 1FQ9) [25]. (**C**) Netrin-1 (Net-1) in complex with sucrose octasulfate (SOS) (PDB ID: 7LRF) [32]. (**D**) Platelet factor 4 (PF4) in complex with the heparin mimetic fondaparinux (PDB ID: 4R9W) [33]. (**E**) Stromal-cell-derived factor 1 (CXCL12) bound to a heparin disaccharide (PDB ID: 2NWG) [34].

Heparan sulfate molecules are important components of multicellular organisms that allow for the correct structural and functional organization of the extracellular matrix. Heparan sulfates interact with various proteins through both electrostatic and hydrogen-bonding interactions [6]. These are characterized by negatively charged sulfate groups on the ligand molecule that are able to interact with positively charged amino acid residues, such as lysine and arginine, creating a strong electrostatic attraction [4]. Additionally, hydrogen-bonding networks can form, which further stabilize the protein–ligand interaction. Heparan sulfate molecules bind to specific sequences of basic amino acids known as Cardin–Weintraub or heparan sulfate/heparin-binding motifs, which are arranged in an appropriate structural conformation [20] and supplemented with other polar residues to improve complex stability [21]. To allow for proper binding, the flexibility, conformation and sulfation patterns of the heparan sulfate molecule are important physicochemical characteristics that determine the specificity and affinity of the ligand (Figure 1). These interactions tend to be very stable, and although the variability of heparan sulfate molecules is very high, binding affinities are in the nanomolar range [22,23,24]. The structural variability of naturally occurring heparan sulfate molecules allows them to mediate the formation and stability of various protein complexes and heterogeneous multimers. As such, heparan sulfates and heparins have been found to interact with growth factors and cell surface receptors, promoting the formation of signaling transduction [25,26]. Furthermore, heparan sulfate and heparin chains also interact with proteins to regulate overall integrity and stability through large protein assemblies [27,28,29]. Over 2800 heparan sulfate binding proteins have been identified as part of the human interactome to date [9]. These proteins form complex protein assemblies in the extracellular matrix that do not necessarily have to serve a function; however, chemical mediation allows for local and temporal regulation of protein content in the extracellular matrix. Thus, the direct and indirect functions of heparan sulfate across the extracellular matrix represent a crucial contribution to the maintenance of tissue integrity and normal physiology [30,31].

One of the key ways in which heparan sulfates directly regulate cellular processes is by modulating the activity of growth factors. Growth factors are signaling molecules that are involved in cell proliferation, differentiation and migration. Heparan sulfates are able to interact with these molecules, promoting the formation of signaling complexes that activate downstream signaling pathways. This is evidenced by the complex formation of FGFR1 and its ligand, which stabilizes the formation of a protein–protein complex [25]. Structural examinations highlight the importance of the heparan sulfate sulfation patterns, as the 6-O-sulfate groups are needed to mediate multiple interactions between the two proteins. Beyond the direct functional aspects, heparan sulfates bind to the heparan sulfate/heparin binding motifs of proteins such as matrix metalloproteinases, interferons and chemokines [35,36,37,38]. These proteins form large multimer assemblies of varying proteins that may not serve a direct function but rather assemble to create specific microenvironments. The assemblies mainly serve a structural purpose that allows the proteins to remain in their proper location upon cell secretion and then form an appropriate and stable extracellular environment around the cell, which can result in the establishment of protein–ligand concentration gradients between cells, which are crucial for homeostasis [39,40,41]. Interestingly, varying ligand concentrations may even result in the activation of opposing signaling cascades; this has been demonstrated for dependence receptors and their appropriate protein ligands [42]. Heparan sulfate molecules take part in these processes, as they restrict free diffusion in the extracellular matrix for proteins containing these binding motifs.

The indirect functional role of heparan sulfate on cellular signaling brings up two possible sources for a wide range of physiological diseases related to uncontrolled signaling. The first is mutations in the direct binding interfaces of proteins that affect ligand binding and subsequent protein diffusion [43]. The crucial amino acids that are associated with the respective Cardin–Weintraub motif in an extracellular matrix protein, such as basic residues for electrostatic attraction, are often associated with disease variants [44]. An example of this is the chemokine Noggin, which has been found to form larger multimer assemblies around heparin chains [18,39]. Mutations in the heparan sulfate/heparin-binding site have been associated with proximal symphalangism [45] and an increase in cell differentiation due to a loss of bone morphogenetic protein (BMP) signaling inhibition [46]. A decrease in heparan sulfate association has been hypothesized to allow for free diffusion of noggin away from the cell surface, which abolishes the control of signaling inhibition [47,48]. Similarly, mutations of the heparan sulfate/heparin-binding sites in the chemokines C-C motif chemokine 2 (CCL2) and CXCL12 were reported to reduce dimer formation and regular chemotactic activity, which impaired downstream signaling events and cell homeostasis [41,49,50].

The second potential cause of disease is irregularities in enzymes that release heparan-sulfate-associated proteins from their structural supports, resulting in free ligand diffusion across the extracellular matrix. Heparanase is an extracellular matrix enzyme that functions to cleave heparan sulfate chains, where abnormal expression and function has been shown to cause uncontrolled cell signaling and remodeling of the extracellular matrix [51,52]. It was demonstrated that upon induced overexpression of Heparanase, human myeloma cells increased in number and size. Interestingly, this effect was reversible upon the addition of C-X-C motif chemokine ligand 10 (CXCL10) [53]. Likewise, increased Heparanase concentrations have been shown to promote tumor growth for glioma, mesothelioma and gastric carcinoma cells [54,55,56]. Although underlying mechanisms are currently not well understood, it is hypothesized that the remodeling of the extracellular matrix and its subsequent inability to regulate heparan-sulfate-bound cytokines and chemokines for cell homeostasis play a key role in disease progression.

In general, heparan-sulfate-mediated protein complex formation plays a critical role in regulating various cellular processes, including cell proliferation, differentiation and migration, by modulating the activity and localization of signaling proteins. Accordingly, the binding and retaining of proteins by heparan sulfates in the extracellular matrix adds a level of complexity to intercellular signaling that is often unaccounted for. As such, the precise mechanisms by which heparan sulfates interact with different proteins to control cellular processes are still not fully understood. Still, our current knowledge of irregularities in the structural integrity of GAG-mediated protein assemblies caused by intrinsic or extrinsic forces emphasizes the significance of heparan sulfate as a structural component. Prospective studies on protein systems in the extracellular matrix should consider possible effects of GAG ligands and the formation of larger protein assemblies that might affect cellular signaling and matrix organization.

## 3. Heparin Mimetics Influence Extracellular Matrix Organization

Heparan sulfate is a crucial component for multicellular life, and it interacts with many different signaling proteins and systems. Although heparan sulfate is a very unspecific ligand due to its overall strong negative charge, various heparin mimetics have been developed that constitute different classes, from basic small saccharides to polysulfated oligosaccharides and non-carbohydrate mimetics [11,57]. Generally, these molecules mimic the structure and function of heparan sulfate or heparin molecules and target the heparan sulfate/heparin-binding motifs or allosteric binding sites of proteins for therapeutic development. Heparin-based drugs are frequently thought of as exclusive anticoagulants; however, recent studies have shown their therapeutic potential in decreasing inflammation and fibrosis, as well as inhibiting cancer progression [11,16,58,59,60].

As heparan sulfates take part in the structurally organization of the extracellular matrix, inflammatory and fibrotic events are highly dependent on the present heparan sulfate composition and function. This includes the careful control of heparan sulfate modeling by enzymes and subsequent formation of protein assemblies that regulate proinflammatory and anti-inflammatory cytokines [49]. This balance can be disrupted by stressors that can then induce disease. For that reason, enzymes that affect matrix remodeling and repair such as Heparanase and human neutrophil elastase (HNE) have been focused on for drug development [12]. The recent design of non-carbohydrate heparin mimetics produced a selective non-competitive inhibitor for HNE and other inflammatory serine proteases that prevent degradation of various extracellular proteins including proteoglycans for structural heparan sulfate support. Inhibition of these pathways would then reduce inflammatory responses. This was confirmed in another study in which the inhibition of Heparanase was linked to inflammation reduction and eventual reduction in cytokine expression levels [61]. Although inhibiting remodeling enzymes is a more indirect approach to regulating protein assemblies, the design of direct heparan sulfate/heparin-binding site inhibitors to alter protein concentration gradients raises other difficulties due to the unspecific binding behaviors of the ligands. However, disruption of protein–heparan sulfate binding could prove to be promising for physiological diseases in which signaling proteins are overexpressed or retained in elevated concentrations around the cell. An example of this is osteoarthritis, in which the extracellular matrix is abnormally remodeled. In a recent study, the GAG content in osteoarthritis patient tissues was examined; results showed that although the total GAG sulfation decreased, heparan sulfate sulfation and overall protein content increased as compared to healthy tissue. Interestingly, binding affinities for influential growth factor proteins decreased by eightfold, which suggests that the sulfation patterns were altered as part of the abnormal extracellular matrix remodeling [62]. These results point into an interesting direction for heparin mimetic drug development, as release of the various proteins and aggregates from the extracellular matrix in osteoarthritis-affected tissues could potentially alleviate irregular cell signaling for effective therapies.

Furthermore, cancer progression is often associated with abnormal remodeling of the immediate extracellular matrix, which allows for elevated cell proliferation and differentiation based on compromised control mechanisms. Synthetic heparin mimetics have recently been produced that show antithrombotic and inhibitory activity of various enzymes such as Heparanase, P-selectin and the integrin VLA-4 to drastically reduce metastatic activity; the heparin mimetic efficiently blocked melanoma cell binding to endothelial cells under blood flowing conditions [16]. This was supplemented by a study that showed successful inhibition of tumor cell migration, invasion and adhesion by a polymer-based heparin molecule via inhibition of Heparanase activity [59]. However, a recent clinical study of the heparin drug tinzaparin presents conflicting results; no significant impact on cancer progression or patient survival was achieved upon drug administration. Although there were some limitations such as heparin-induced thrombocytopenia, results suggest limited effects of tinzaparin on early-stage cancer growth [63]. Still, as heparin-mimetic drug developments have mainly focused on the metastatic repression of cancers [15,64,65], this study provides valuable information on the limitations of low-molecular-weight heparins as clinical drugs. The recent advancements of more complex synthetic heparin mimetics demonstrate compelling non-anticoagulant abilities for disease treatments. Hence, the direct inhibition of enzyme activity and cellular signaling by heparin mimetics are still a promising avenue for antimetastatic drug development.

**Figure 2:**
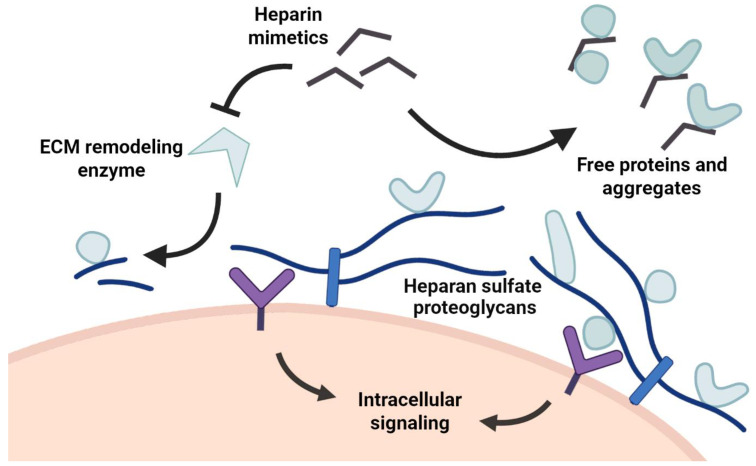
Heparin mimetics affect various biochemical mechanisms in the extracellular matrix. Heparin mimetics inhibit extracellular remodeling enzymes such as Heparanase and impact regular remodeling pathways and structural integrity. Heparin mimetics can also bind to heparan sulfate binding proteins and change protein concentration levels near the cell surface. Both of these mechanisms cause an eventual change in intracellular signaling, which might affect cell homeostasis.

Beyond the direct effects of heparin mimetics on enzymes and signaling proteins, changes that affect general protein–heparan sulfate interactions and protein assemblies can alter signaling behavior. An example of this is the antagonist removal from the immediate surroundings of a cell surface [41,66], which can be induced by free heparin-based molecules that outcompete regular heparan sulfate binding [67,68]. This would result in free diffusion of the signaling protein and dissolve concentration gradients, resulting in serious consequences, as biochemical regulation mechanisms would be lost. Nonetheless, increased stability of heparan sulfate-bound proteins has also been shown to affect signaling; biomaterials that contained heparin were able to induce osteogenesis by stabilizing BMP signaling proteins around cellular surfaces [69]. Studies such as these illustrate the importance of appropriate heparan sulfate modeling, which regulates the structural stability and integrity of protein assemblies. On one hand, the disruption of protein assemblies in the extracellular matrix has significant consequences on localized ligand concentrations for cell signaling, while excessive protein assemblies and stability cause overstimulation of signaling pathways. Heparin mimetics in combination with nanomaterials therefore offer a wide therapeutic range for various diseases.

In any case, the underlying disease mechanisms in relation to heparan sulfate or heparin dependence have to be examined in order for appropriate drug development to occur. Heparin mimetics can function as inhibitors against extracellular matrix deconstruction and remodeling (Figure 2) [16], as well as directly disrupting the structural organization of protein assemblies [8,60]. Depending on the desired outcome, different biochemical systems have to be targeted, which requires an adequate understanding of the interacting components in a given signaling system. This would also support more effective structural based drug design for improved heparin mimetics that precisely target specific protein interaction surfaces. Novel drug candidates should aim to reduce anticoagulant activity to minimize negative side effects by improving specificity. Here, a more specific drug would have features that match the target’s natural binding to heparan sulfate; these include appropriate sulfation patterns, shape, length and flexibility, as well as functional groups for hydrogen bond formation. Although we have a general understanding of how heparan sulfates interact with proteins, the specific structural details for individual proteins and the overall functional contexts of cellular signaling are currently under-represented. To improve our understanding of the extracellular matrix and the development of heparin mimetics for therapeutic approaches, heparan sulfate binding should be part of regular investigations, especially in studies that examine extracellular protein signaling systems.

## Data Availability

All the relevant data is contained within this article.
